# Effect of Social Interaction Intervention on the Elderly:A Systematic Review

**DOI:** 10.1002/alz.088015

**Published:** 2025-01-09

**Authors:** Soo Yeon Yoo, Jung Min Han, Ji‐Hyuk Park

**Affiliations:** ^1^ Yonsei university, Wonju‐si, Gangwon‐do Korea, Republic of (South); ^2^ Yonsei University, Wonju, Gangwon‐do Korea, Republic of (South)

## Abstract

**Background:**

Exercise or cognitive interventions have already been sufficiently provided to the elderly, and their effectiveness has been proven. However, interventions in the context of promoting social interaction have not been well‐implemented for the elderly, and few studies have considered the effectiveness of interaction mediation for the elderly. This study therefore sought to examine the effectiveness of interactive interventions by examining studies that provide interventions focusing on social interactions.

**Methods:**

We conducted a systematic search spanning the period from 2013 to 2023, utilizing databases such as RISS, DBpia, Embase, and Web of Science. The primary search terms employed were (“elderly” OR “geriatric”) AND “social interaction” AND intervention.” Four carefully selected studies were subjected to detailed analysis.

**Result:**

Among the four selected studies, three specifically evaluated cognitive function and confirmed the results. The interventions demonstrated a notable increase in both social interaction and cognitive function, coupled with a decrease in depression.

**Conclusion:**

This study examined interventions targeting social interaction among older adults. These findings serve as foundational data for designing effective social interaction interventions within the context of occupational therapy for the elderly.

